# Case Report: Scrub Typhus manifesting as Acute Respiratory Distress Syndrome (ARDS) with corresponding radiological findings

**DOI:** 10.12688/f1000research.141986.1

**Published:** 2023-12-15

**Authors:** Jay Dinesh Bhanushali, Babaji Ghewade, Ulhas Jadhav

**Affiliations:** 1 Respiratory Medicine, Jawaharlal Nehru Medical College, Sawangi Meghe, Wardha, Maharashtra, 442001, India

**Keywords:** Scrub typhus, acute respiratory distress syndrome, ARDS, computed tomography, CT scan

## Abstract

**Background:**

Scrub typhus is a life-threatening infectious disease endemic in the Asia-Pacific region. It typically presents with nonspecific symptoms such as fever, headache, and myalgia, making early diagnosis challenging. Acute Respiratory Distress Syndrome (ARDS) is a severe pulmonary condition characterized by acute-onset hypoxemia, bilateral lung infiltrates on radiology, and increased pulmonary capillary permeability.

**Case Presentation:**

An 18-year-old female student in central India presented with a seven-day history of recurrent fever, chills, dry cough, and severe shortness of breath, escalating to Modified Medical Research Council dyspnea grade III-IV. After unsuccessful local clinic treatment, a chest radiograph revealed bilateral pneumonia. On admission, she displayed tachycardia, tachypnea, hypotension, and hypoxia requiring non-invasive ventilation (NIV). Computed tomography confirmed scrub typhus-associated pneumonia, and serological testing was positive for scrub typhus. She was diagnosed with moderate ARDS and began treatment. Symptomatic improvement was seen in the ICU, and she was discharged on day 10 with radiological and clinical resolution.

**Management and Outcomes:**

She received intravenous doxycycline and oral azithromycin for scrub typhus and any potential concurrent lung infection. In the ICU, she required continuous NIV and supplemental oxygen, with significant symptomatic improvement, evidenced by reduced tachypnoea and oxygen requirements after 72 hours. She was weaned off NIV and monitored for an additional four days. After satisfactory oxygen saturation on room air, she was discharged on the tenth day. High-resolution CT scan demonstrated resolution of ground glass opacities and consolidation. Sequential chest radiographs exhibited gradual reduction in bilateral alveolar infiltrates over time, in parallel with clinical improvement. Laboratory findings, including reduced CRP and D-dimer values, and a normal hemogram on discharge indicated a resolution of leukopenia.

**Conclusion:**

This case underscores the importance of early recognition and intervention in scrub typhus-associated ARDS and highlights the utility of timely diagnostic imaging in monitoring the progress of the disease.

## Introduction

Scrub typhus, an infectious ailment brought on by the rickettsial microbe Orientia tsutsugamushi, presents a significant challenge to public health across the Asia-Pacific region. It’s notably prominent within the expansive domain referred to as the “tsutsugamushi triangle.” ^1^^,^^2^ Notably, a recent study on the burden of scrub typhus in India, situated within this geographical region, unveiled its substantial impact, contributing to at least 25.3% of acute undifferentiated febrile illness (AUFI) cases. This disease typically exhibits clinical symptoms following an incubation period of 6-21 days, characterized by fever, headaches, myalgia, and various gastrointestinal issues. ^3^

The pathophysiology of O. tsutsugamushi involves the infection of endothelial cells, leading to vasculitis, followed by the infiltration of T cells and monocytes/macrophages around blood vessels. This sets off a complex cascade of inflammatory responses, where various cytokines are produced by both endothelial and non-endothelial cells. These responses, while serving antimicrobial purposes, can also harm the host’s tissues. This dual nature of the immune response can give rise to severe complications, including hepatitis, renal failure, meningoencephalitis, acute respiratory distress syndrome (ARDS), respiratory failure, and occasionally myocarditis. ^1^

Typical clinical presentations of scrub typhus encompass fever, generalized lymph node enlargement, cough, shortness of breath, muscle pain, headaches, skin rashes, eschar formation, loss of appetite, vomiting, and abdominal discomfort. Unusual complications, although infrequent, may include meningoencephalitis, glomerulonephritis, acute renal failure (ARF), interstitial pneumonia, acute respiratory distress syndrome (ARDS), acute hepatic failure, myocarditis, pericarditis, gastrointestinal bleeding, septic shock, acute hearing loss, acute cholecystitis, and intracranial hemorrhage. ^4^

Scrub typhus manifesting as Acute Respiratory Distress Syndrome (ARDS) is unique in medical literature due to the convergence of a tropical infectious disease, caused by Orientia tsutsugamushi, with a severe respiratory condition. This combination is distinctive because it involves a broad spectrum of clinical complications, which can include life-threatening ARDS, not commonly associated with tropical infections. The rarity of this manifestation and the complex interplay between vasculitis, immune responses, and tissue damage make it a subject of special interest in the medical community. Understanding this unique presentation is crucial for early diagnosis and effective management, given the potential for severe morbidity and mortality associated with scrub typhus-induced ARDS. ^5^

## Case report

On the 9th of October 2022, an 18-year-old student hailing from Wardha, India, with recent travel history to Amravati, was urgently brought to Acharya Vinoba Bhave Hospital. She presented with a triad of fever accompanied by chills and rigors, recurring over the past week. In addition to her febrile episodes, she reported a persistent dry cough for three days and experienced a notable exacerbation of shortness of breath over the previous two days, reaching a severity classified as grade III–IV on the modified Medical Research Council (mMRC) dyspnea scale. Despite seeking initial treatment at a local clinic for two days, her symptoms showed no improvement. Subsequently, a chest radiograph revealed bilateral pneumonia, prompting her referral to the hospital for comprehensive evaluation and management.

During her initial hospitalization, the patient continued to suffer from fever, chills, and acute shortness of breath. She experienced orthopnoea (breathlessness when lying down), and her dry cough exacerbated when supine. On admission, her vital signs included a high pulse rate of 127 beats per minute, a rapid respiratory rate of 36 breaths per minute, low blood pressure at 100/70 mmHg, and a body temperature of 98.7°C. A respiratory examination revealed diminished breath sounds bilaterally and scattered crepitations in both lung fields. No evident abnormalities were observed in her central nervous, cardiovascular, or gastrointestinal systems.

Arterial blood gas analysis (ABG) confirmed type I respiratory failure with severe hypoxia on room air, necessitating non-invasive positive pressure ventilation. The patient’s PF ratio of 153 mmHg suggested moderate ARDS as per Berlin’s definition. A chest CT upon admission revealed diffuse ground glass opacities with patchy consolidations in both lungs, indicative of interstitial pneumonia, along with bilateral mild pleural effusion and a CT Severity index of 21/25, illustrating disease progression. These findings were consistent with characteristic imaging manifestations of scrub typhus-associated pneumonia.

A complete blood count demonstrated leukopenia with a white blood cell count of 3200 cells/mm ^3^, and smears and antigen testing for malarial parasites were negative. Blood samples were forwarded for further analysis, and the initial treatment regimen included intravenous piperacillin and tazobactam (4.5 grams three times a day) alongside azithromycin (500 mg once daily). The patient also received nebulized bronchodilators and inhaled corticosteroids.

The diagnostic workup, focusing on the patient’s fever pattern, confirmed a positive serological test for scrub typhus while testing negative for Widal, leptospira, and dengue. Blood and sputum samples, despite being sent for culturing, showed no growth. Elevated levels of C-reactive protein and D-dimer, notably CRP 108 and D-dimer 2350, led to the initiation of low molecular weight heparin therapy. A diagnosis of scrub typhus with moderate ARDS was established, prompting the initiation of intravenous doxycycline (100 mg twice daily) and oral azithromycin (500 mg once daily) to manage any concomitant lung infection.

In the ICU, the patient required noninvasive ventilation (NIV) for the initial four days continuously, supplemented with 8-10 L of oxygen via a face mask during meals. She exhibited symptomatic improvement after 72 hours, with a gradual reduction in tachypnea and a decreased need for oxygen. Consequently, she was successfully weaned off NIV and transferred to the general ward, where she was monitored for an additional four days. Her ability to maintain oxygen saturation on room air improved, eliminating the need for external oxygen support, and she was discharged on day 10 of her hospital stay.

**Figure 1.**
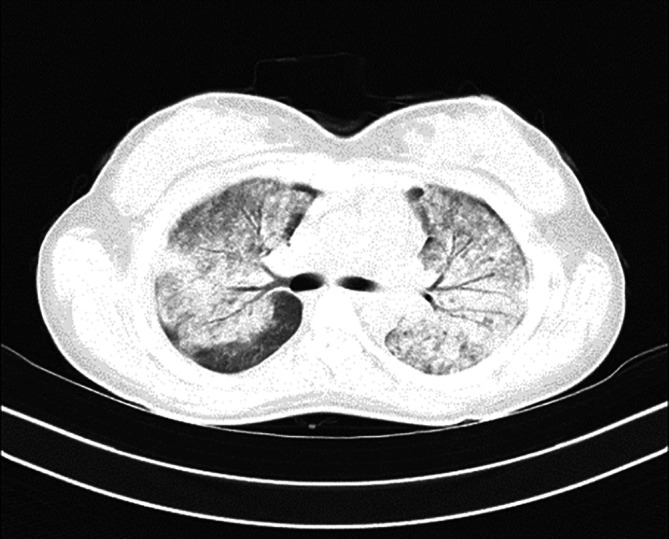
On admission, HRCT of the thorax shows bilateral extensive diffuse areas of ground glass opacification with superimposed patchy consolidation; with a CT severity score of 21/25. These findings were consistent with the characteristic imaging manifestations of scrub typhus-associated ARDS.

**Figure 2.**
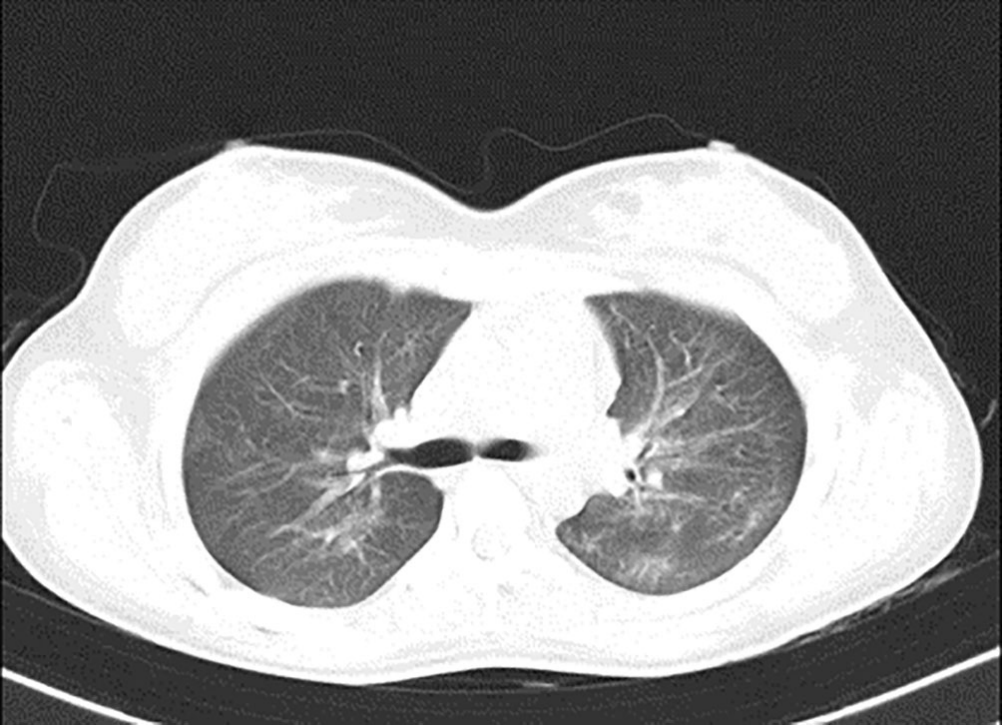
On discharge, HRCT Thorax suggestive of good resolution with non-specific scattered ground glass opacities and no evidence of fibrosis.

**Figure 3.**
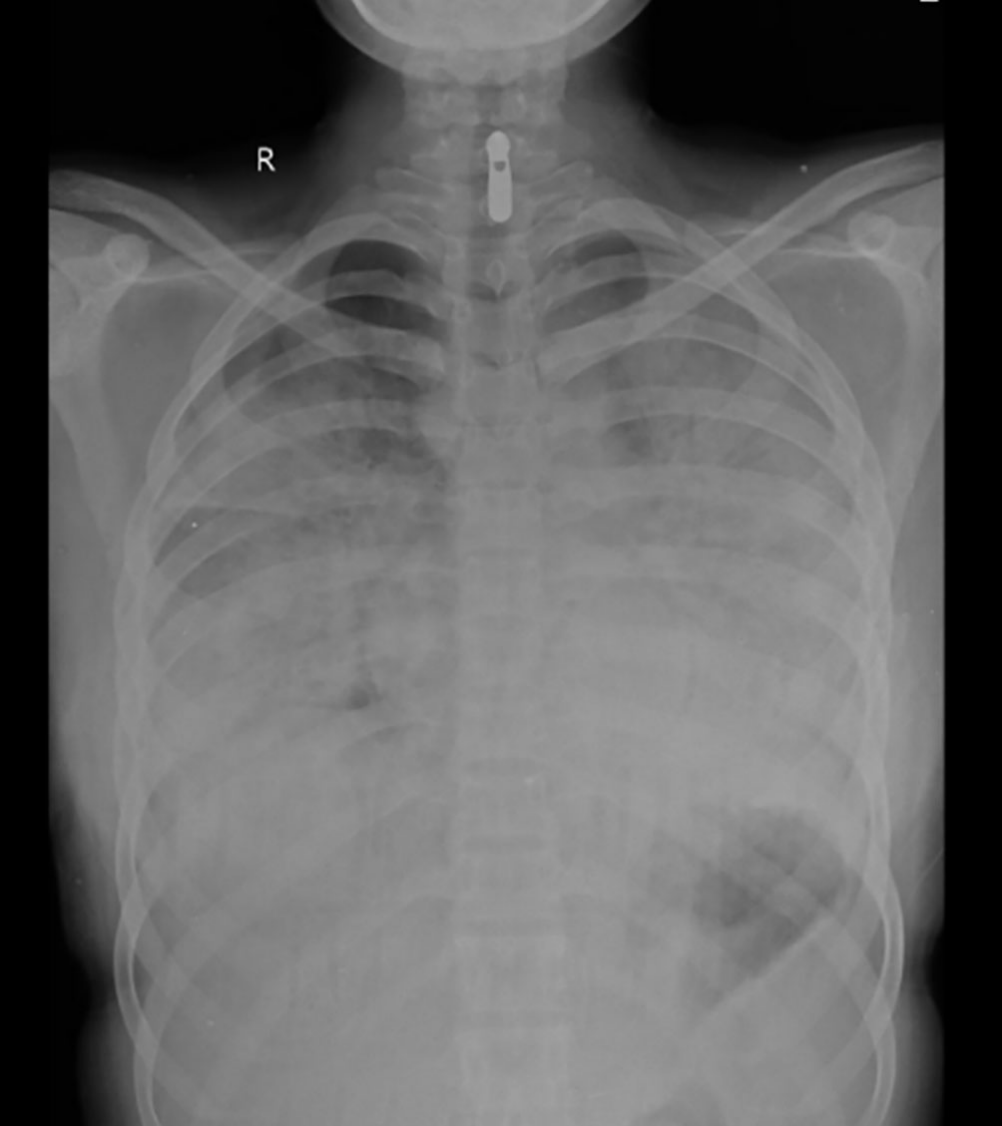
Chest radiograph PA view, day 1.

**Figure 4.**
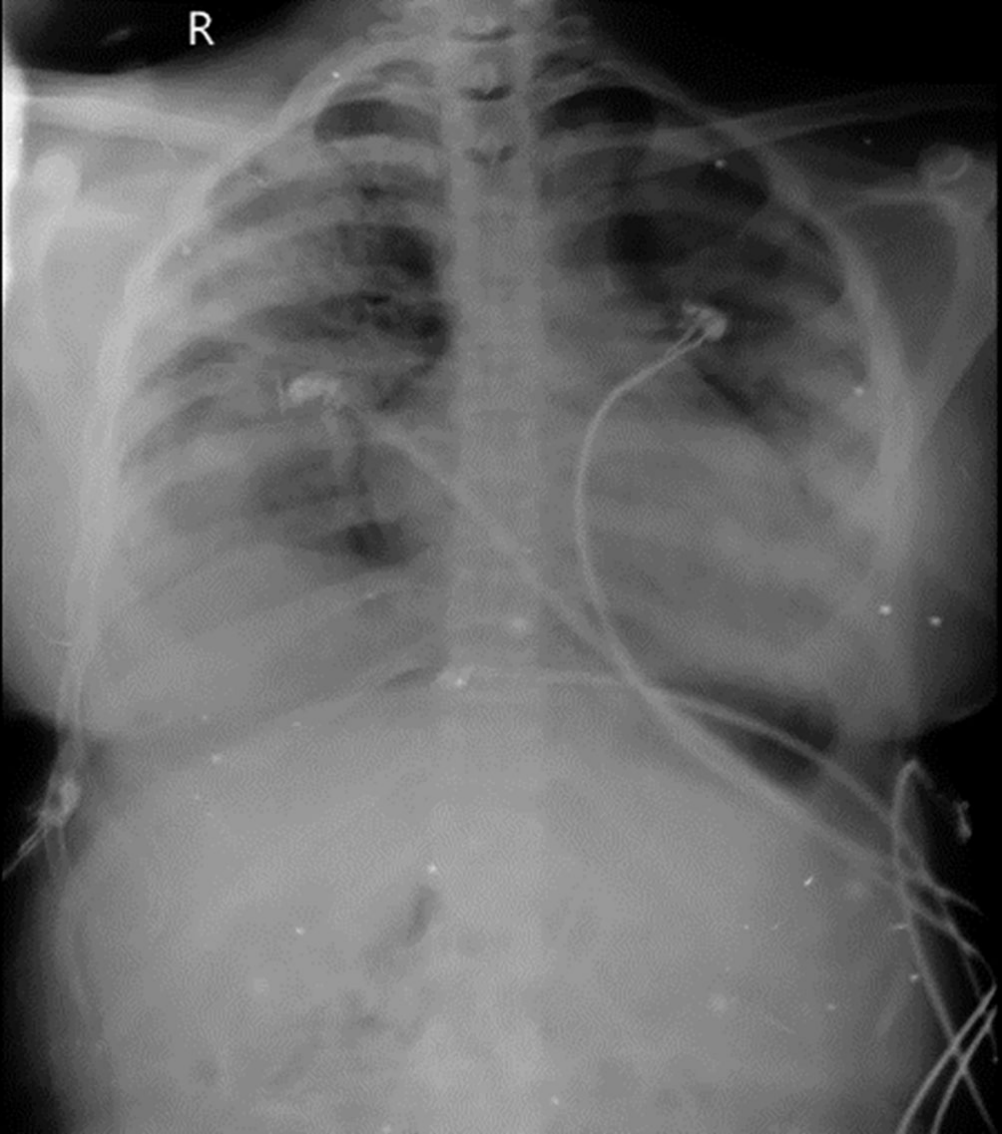
Chest radiograph PA view, day 5.

**Figure 5.**
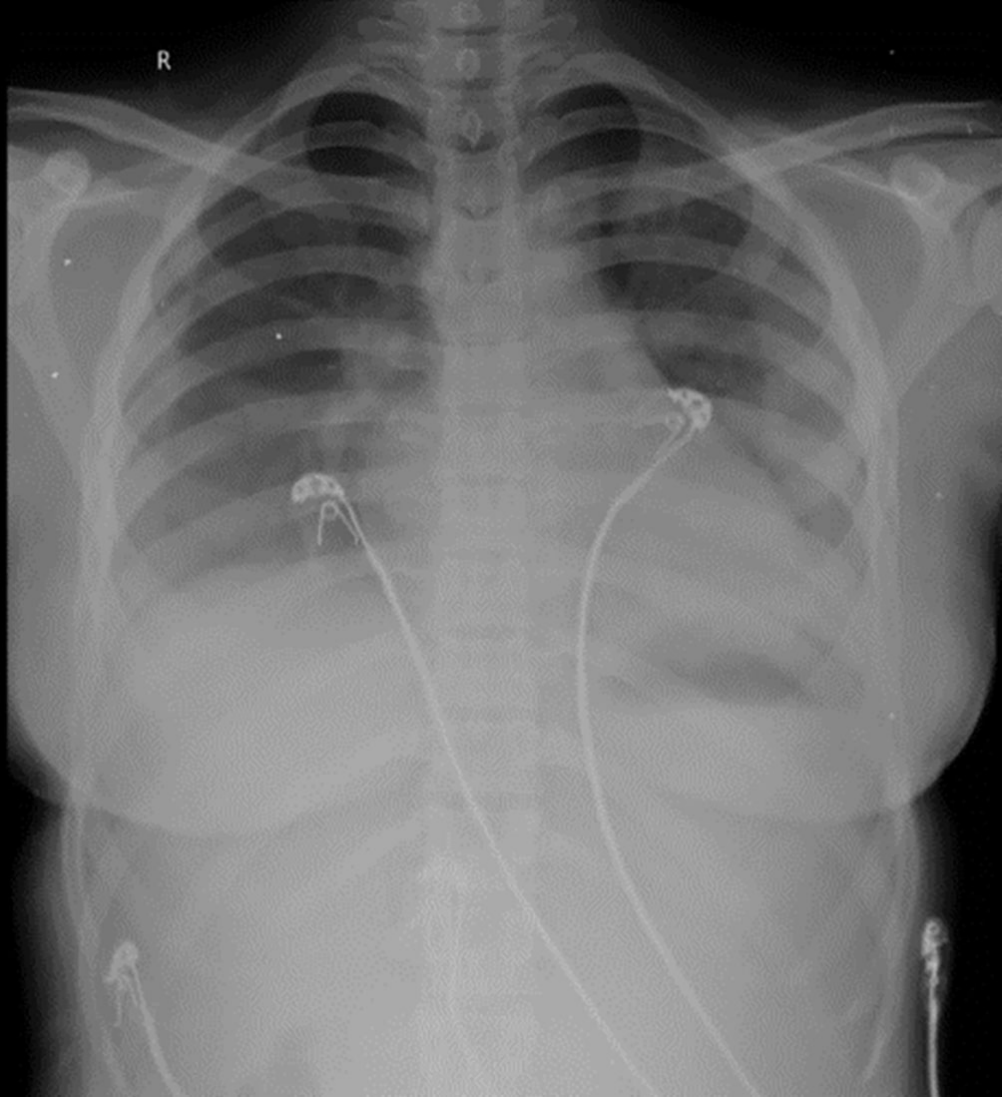
Chest radiograph PA view, day 8.

In Figure 1, upon admission, the high-resolution computed tomography (HRCT) of the thorax reveals bilateral extensive diffuse areas of ground glass opacification, accompanied by superimposed patchy consolidation. The CT severity score for these findings is notably high, with a recorded score of 21 out of 25. These radiological characteristics align with the typical imaging manifestations of scrub typhus-associated acute respiratory distress syndrome (ARDS). High-resolution chest CT scan conducted before discharge illustrated the satisfactory resolution of ground glass opacities and consolidations, affirming her clinical improvement ( Figure 2). Sequential chest radiographs, as shown in Figures 3, 4, and 5 displayed a gradual reduction in bilateral alveolar infiltrates over time, further correlating radiological resolution with symptomatic improvement. These positive outcomes were reinforced by significant changes in diagnostic markers during her follow-up, where CRP and D-dimer values markedly reduced and her hemogram returned to normal, demonstrating the resolution of leukopenia. CRP decreased from 108 to 21, D-dimer decreased from 2350 to 430, hemoglobin slightly decreased from 11.5 to 11.2, white blood cell count increased from 3200 to 4900, and platelet count improved from 245,000 to 345,000. This comprehensive case presentation underscores the patient’s challenging journey from illness to recovery, emphasizing the vital role of early diagnosis and effective management in scrub typhus-associated ARDS.

## Discussion

Scrub typhus is a prevalent infectious disease in India, with a higher susceptibility among individuals residing in rural areas, particularly agricultural workers and military personnel. ^6^^,^^7^ The clinical spectrum of scrub typhus varies from constitutional symptoms commonly seen in acute febrile illnesses, such as fever, headache, cough, and the presence of an eschar, to severe and potentially life-threatening complications, including pneumonitis, encephalopathy, shock, and septicemia. ^8^ The characteristic necrotic eschar associated with scrub typhus is typically observed at the site of the wound. Fatality rates are influenced by delayed presentation, diagnostic challenges, and drug resistance. ^8^ Importantly, scrub typhus has the potential to progress into acute respiratory distress syndrome (ARDS), displaying a spectrum of lung-related symptoms ranging from bronchitis and interstitial pneumonitis to the eventual onset of ARDS. ^9^ In a comprehensive study by Wang CC et al., which focused on ARDS among individuals afflicted with scrub typhus, a mortality rate of approximately 25% was reported. However, it’s noteworthy that this figure could be mitigated through the prompt application of appropriate interventions, such as doxycycline or chloramphenicol. ^10^

In the presented case, the absence of a pathognomonic eschar posed a diagnostic challenge, necessitating the diagnosis of scrub typhus primarily based on ELISA results. Notably, this diagnostic approach underscores the significance of considering scrub typhus as a potential etiology for ARDS, especially in endemic regions.

The identification of diffuse bilateral ground-glass opacities with a peripheral and subpleural distribution, consolidations, and mediastinal lymphadenopathy on CT scans can contribute to the diagnosis of scrub typhus-associated pneumonia. In this case, timely recognition and the initiation of appropriate antibiotic therapy were critical for enhancing patient outcomes.

The strengths of our approach lie in the prompt recognition of scrub typhus as a potential cause of ARDS, even in the absence of a characteristic eschar. The utilization of diagnostic imaging, such as CT scans, played a pivotal role in reaching an accurate diagnosis. Additionally, the adherence to evidence-based treatment protocols, including the use of appropriate antibiotics and non-invasive positive pressure ventilation, demonstrated positive outcomes for the patient.

However, there are some limitations to consider. The delayed presentation of the patient to the healthcare facility highlights the need for increased awareness and early diagnostic strategies for scrub typhus. This case emphasizes the significance of early diagnosis, as delays can lead to severe complications. Additionally, despite the positive outcome in this case, it is essential to acknowledge that scrub typhus-associated ARDS can be associated with high mortality, making early intervention and accurate diagnosis crucial.

The follow-up diagnostic information in this case, including the significant reduction in CRP and D-dimer values and the return to normal hemogram, indicates the resolution of leukopenia and demonstrates the effectiveness of the treatment. The patient’s perspective on the use of Bipap is noteworthy, as it reflects the importance of patient compliance and adaptation to treatment measures. The motivation and growing confidence of the patient with noticeable symptomatic improvement and reduced respiratory effort underscore the collaborative role of both clinicians and patients in achieving positive outcomes.

## Conclusion

In conclusion, this case highlights the multifaceted challenges and successes in the diagnosis and management of scrub typhus-associated ARDS. It underscores the importance of early recognition, diagnostic imaging, and timely intervention in endemic regions, and the potential for positive outcomes when evidence-based treatment protocols are adhered to. Patient outcomes, as demonstrated in this case, underscore the significance of patient cooperation and adaptation to treatment measures, contributing to improved recovery and overall success in managing scrub typhus-associated ARDS.

## Consent

Written informed consent from the patient for the use and publication of patient’s data was taken.

## Data Availability

All data underlying the results are available as part of the article and no additional source data are required.
