# Supplementary material for: Case Report: Scrub Typhus manifesting as Acute Respiratory Distress Syndrome (ARDS) with corresponding radiological findings
Source: F1000Res. 2024 Apr 29;12:1596. Originally published 2023 Dec 15. [Version 2] doi: 10.12688/f1000research.141986.2 (PMC11399754; doi:10.12688/f1000research.141986.2)
Supplement: Peer review history

# Reviewer report: Reviewer response for version 2

**DOI:** 10.5256/f1000research.165517.r317962

**Published:** 2024-09-27

**Authors:** Susmitha Karunasree Perumalla

**Affiliations:** 1 Christian Medical College Vellore Association, Vellore, Tamil Nadu, India

The case report has relevant information on ARDS in scrub typhus.

 Regarding the background of the case, no information on prior treatment before the patient came to the current health care setting was mentioned. Specifically history of any antibiotics used outside, as this could reiterate the role of right group of antibiotics in treatment of scrub typhus. 

 Since it is a case of scrub typhus and the supporting evidence clearly here is the serological testing, it will be worthwhile elaborating on the ELISA (IgM/IgG, kit name and the target antigen used).

Are enough details provided of any physical examination and diagnostic tests, treatment given and outcomes?

Yes

Is the case presented with sufficient detail to be useful for other practitioners?

Yes

Is sufficient discussion included of the importance of the findings and their relevance to future understanding of disease processes, diagnosis or treatment?

Yes

Is the background of the case’s history and progression described in sufficient detail?

Partly

Reviewer Expertise:

Zoonoses, Immunodiagnostics of infectious and auto immune conditions

I confirm that I have read this submission and believe that I have an appropriate level of expertise to confirm that it is of an acceptable scientific standard.

# Reviewer report: Reviewer response for version 2

**DOI:** 10.5256/f1000research.165517.r270079

**Published:** 2024-09-13

**Authors:** Zahid Khan

**Affiliations:** 1 Barts Health NHS Trust, London, UK; 2 Cardiology, University of South Wales, Pontypridd, Wales, UK; 3 Cardiology, The University of Buckingham, Buckingham, England, UK

Case report section. Please change this from (high pulse rate of 127 beats per minute regular rhythm to (sinus tachycardia with a HR of 127 bpm, tachypneoa with a RR of 36).

 A complete blood count demonstrated leukopenia with a white blood cell count of 3200 cells/mm ^3^ (Please include reference value)

 Please use arrows to highlight findings in these images.

 Dear authors/Editor,

 There is improvement in this article and I have suggested a few minor changes. I have not included any reservation as these are minor changes that can be made to avoid this cycle of repeating reviews.

 Many thanks,

 Zahid

Are enough details provided of any physical examination and diagnostic tests, treatment given and outcomes?

Yes

Is the case presented with sufficient detail to be useful for other practitioners?

Yes

Is sufficient discussion included of the importance of the findings and their relevance to future understanding of disease processes, diagnosis or treatment?

Yes

Is the background of the case’s history and progression described in sufficient detail?

Yes

Reviewer Expertise:

Cardiology, Respiratory Medicine, GIM and Gastroenterology.

I confirm that I have read this submission and believe that I have an appropriate level of expertise to confirm that it is of an acceptable scientific standard.

# Reviewer report: Reviewer response for version 2

**DOI:** 10.5256/f1000research.165517.r270078

**Published:** 2024-05-14

**Authors:** Philippe Jouvet, Arnaud Wiedmann

**Affiliations:** 1 University of Montréal, Montréal, Canada; 2 Pediatrics, CHU Nancy, Nancy, France; 3 Pediatrics, Sainte-Justine Hospital, Montreal, Québec, Canada

Bhanushali et al. completed the informations related to the clinical case as best as possible. The case described enriches rare literature on the topic, making it possible to serve as a basis for discussion for similar situations.

Are enough details provided of any physical examination and diagnostic tests, treatment given and outcomes?

Yes

Is the case presented with sufficient detail to be useful for other practitioners?

Yes

Is sufficient discussion included of the importance of the findings and their relevance to future understanding of disease processes, diagnosis or treatment?

Yes

Is the background of the case’s history and progression described in sufficient detail?

Yes

Reviewer Expertise:

Pediatric Intensive care

We confirm that we have read this submission and believe that we have an appropriate level of expertise to confirm that it is of an acceptable scientific standard.

# Reviewer report: Reviewer response for version 1

**DOI:** 10.5256/f1000research.155477.r259630

**Published:** 2024-04-04

**Authors:** Zahid Khan

**Affiliations:** 1 Barts Health NHS Trust, London, UK; 2 Cardiology, University of South Wales, Pontypridd, Wales, UK; 3 Cardiology, The University of Buckingham, Buckingham, England, UK

Please use the full terminology for all abbreviations when using first time. *O. tsutsugamushi * can be used in the first sentence in the introduction section after *Orientia tsutsugamushi*.

 This disease typically exhibits clinical symptoms following an incubation period of 6-21 days, characterized by fever, headaches, myalgia, and various gastrointestinal issues **.**

 (Please provide frequency of these symptoms)

 Typical clinical presentations of scrub typhus encompass fever, generalized lymph node enlargement, cough, shortness of breath, muscle pain, headaches, skin rashes, eschar formation, loss of appetite, vomiting, and abdominal discomfort. Unusual complications, although infrequent, may include meningoencephalitis, glomerulonephritis, acute renal failure (ARF), interstitial pneumonia, acute respiratory distress syndrome (ARDS), acute hepatic failure, myocarditis, pericarditis, gastrointestinal bleeding, septic shock, acute hearing loss, acute cholecystitis, and intracranial hemorrhage. **(**Please provide frequency for these complications or clinical features)

 In the case report section, please avoid providing specific date and time of the presentation to minimize the risk of patient identification.

 The vitals need further clarity and I think the temperature presented is wrong. It can not be 98C. Heart rate of 127 ? sinus tachycardia?

 Please include reference for the lab results used in the case report.

 The patient also received nebulized bronchodilators and inhaled corticosteroids.(What was the reason for this?

 Did this patient have any ABG prior to starting on NIV? Please include the results in the case report. 

 Please consider using arrows to highlight findings or areas of interest in the images. 

 The case report is well written however needs some changes  as highlighted in the report. It provides details about a health problem of significant importance.

Are enough details provided of any physical examination and diagnostic tests, treatment given and outcomes?

Yes

Is the case presented with sufficient detail to be useful for other practitioners?

Yes

Is sufficient discussion included of the importance of the findings and their relevance to future understanding of disease processes, diagnosis or treatment?

Yes

Is the background of the case’s history and progression described in sufficient detail?

Yes

Reviewer Expertise:

Cardiology, Respiratory Medicine, GIM and Gastroenterology.

I confirm that I have read this submission and believe that I have an appropriate level of expertise to confirm that it is of an acceptable scientific standard, however I have significant reservations, as outlined above.

# Response

**Published:** 2024-04-24

**Authors:** Jay Bhanushali

Thank You for your repose and highlighting the important modifications. Same have been done,

 Please use the full terminology for all abbreviations when using first time. *O. tsutsugamushi * can be used in the first sentence in the introduction section after *Orientia tsutsugamushi*.

 - Changes done

 This disease typically exhibits clinical symptoms following an incubation period of 6-21 days, characterized by fever, headaches, myalgia, and various gastrointestinal issues **.**

 (Please provide frequency of these symptoms)

 - Our patient gave history of only fever with chills and rigors. Denied any other symptoms. 

 In the case report section, please avoid providing specific date and time of the presentation to minimize the risk of patient identification.

 - changes made

 The vitals need further clarity and I think the temperature presented is wrong. It can not be 98C. Heart rate of 127 ? sinus tachycardia?

 - Changes made.

 - Tachycardia + Rhythm regular

 Please include reference for the lab results used in the case report.

 - added

 The patient also received nebulized bronchodilators and inhaled corticosteroids.(What was the reason for this?

 - patient had episodic wheezing hence bronchodilators and ICS added. No past history of OAD.

 Did this patient have any ABG prior to starting on NIV? Please include the results in the case report. 

 - Yes, Details added

# Reviewer report: Reviewer response for version 1

**DOI:** 10.5256/f1000research.155477.r243744

**Published:** 2024-02-22

**Authors:** Philippe Jouvet, Arnaud Wiedmann

**Affiliations:** 1 University of Montréal, Montréal, Canada; 2 Pediatrics, CHU Nancy, Nancy, France; 3 Pediatrics, Sainte-Justine Hospital, Montreal, Québec, Canada

Bhanushali and al. report a case of a 18 year-old indian female, who presented an ARDS due to scrub typhus. Scurub typhus is a frequent cause of unspecific acute undifferentiated febrile illness (fever, headaches, myalgia, and various gastrointestinal issues) in the Asia-Pacific region, due to the rickettsial microbe *Orientia tsutsugamushi*.

*O. tsutsugamushi* infecte endothelial cells responsible of inflammatory cascade activation, which is involved in unusual manifestations such as hepatitis, meningoencephalitis, myocarditis or ARDS.

 According to authors, ARDS is a rare complication in case of tropical infectious disease, whereas its frequently observed in case of *O. tsutsugamushi* infection, due to this inflammatory activation. The different figures offer a useful view of lungs evolution according to time, which is often discuss and debate by clinicians. This manuscript is well written but some additional information are needed.

 Comments.

- The patient presents a bilateral pneumonia, observed in chest radiography and in chest CT. There was revealed diffuse ground glass opacities with patchy consolidations in both lungs, indicative of interstitial pneumonia. Authors report in the abstract and the description of the case that it is “characteristic imaging manifestations of scrub typhus-associated pneumonia”. Why non-specific interstitial pneumonia images are characteristic of scrub typhus-associated pneumonia? Can the authors further specify what are the differential diagnosis ?
- Authors report a “low blood pressure at 100/70 mmHg”, and a “body temperature of 98.7°C”.  A blood pressure at 100/70 mmHg is in the normal range, maybe there was other shock signs such as decrease urinary output? A temperature of 98.7°C is not compatible with life. Did authors mean 38.7°C?
- The authors report a neutropenia but no neutrophils count is provided (only white blood cells)
- Authors only report the initial PaO _2_FiO _2_ ratio (at 153mmHg). They only notice the initial management with Non-Invasive Ventilation, without information in FiO _2_, or PEEP value, or PEEP evolution compared to chest radiography evolution. Could they had such information may be with a graph?
- Initial antimicrobial therapy was intravenous piperacillin and associated to azithromycin. Is it due to antimicrobial resistance profile of bacteria? Even in case of interstitial pneumonia images, which is due to other bacterias than community-acquired pneumonia?
- Why introduction of nebulized bronchodilators and inhaled corticosteroids treatment? There are not recommended of ARDS, may it an interesting treatment in case of *O. tsutsugamushi *pulmonary infection?
- Low molecular weight heparin therapy seems to have been introduced according to the existence of a pro-inflammatory status (Elevated levels of C-reactive protein and D-dimer). With a NIV support responsible of a bed rest in a pubescent patient, is it not the standard care? Or is it a specific treatment in case of severe *O. tsutsugamushi* infection?
- The conclusion could reinforce that Interstitial pneumonia images should make perform an ELISA test to search scrub typhus and introduce appropriate antibiotic treatment early in case of documentation and in such cases the treatment of *O. tsutsugamushi *is based on doxycycline or chloramphenicol, which are not the standard treatment in case of pneumonia.

Are enough details provided of any physical examination and diagnostic tests, treatment given and outcomes?

Yes

Is the case presented with sufficient detail to be useful for other practitioners?

Yes

Is sufficient discussion included of the importance of the findings and their relevance to future understanding of disease processes, diagnosis or treatment?

Yes

Is the background of the case’s history and progression described in sufficient detail?

Yes

Reviewer Expertise:

Pediatric Intensive care

We confirm that we have read this submission and believe that we have an appropriate level of expertise to confirm that it is of an acceptable scientific standard, however we have significant reservations, as outlined above.

# Response

**Published:** 2024-04-24

**Authors:** Jay Bhanushali

Thank you for your detailed review of our case report. 

 We appreciate you highlighting the important points and have made the necessary changes.

# Response

**Published:** 2024-04-24

**Authors:** Jay Bhanushali

1. The patient presents a bilateral pneumonia, observed in chest radiography and in chest CT. There was revealed diffuse ground glass opacities with patchy consolidations in both lungs, indicative of interstitial pneumonia. Authors report in the abstract and the description of the case that it is “characteristic imaging manifestations of scrub typhus-associated pneumonia”. Why non-specific interstitial pneumonia images are characteristic of scrub typhus-associated pneumonia? Can the authors further specify what are the differential diagnosis ? *Changes made, Yes I agree ,scrub typhus was a serological diagnosis, cannot confirm the same of CT scan*
2. Authors report a “low blood pressure at 100/70 mmHg”, and a “body temperature of 98.7°C”.  A blood pressure at 100/70 mmHg is in the normal range, maybe there was other shock signs such as decrease urinary output? A temperature of 98.7°C is not compatible with life. Did authors mean 38.7°C?
*Typing error, also I confirm blood pressure was normal and not low , changes made*
3. The authors report a neutropenia but no neutrophils count is provided (only white blood cells)
*Patient had leucopenia not neutropenia in particular.*
4. Authors only report the initial PaO _2_FiO _2_ ratio (at 153mmHg). They only notice the initial management with Non-Invasive Ventilation, without information in FiO _2_, or PEEP value, or PEEP evolution compared to chest radiography evolution. Could they had such information may be with a graph? 
*Initial settings of NIV added to the report, graph is not available as data was not recorded.*
5. Initial antimicrobial therapy was intravenous piperacillin and associated to azithromycin. Is it due to antimicrobial resistance profile of bacteria? Even in case of interstitial pneumonia images, which is due to other bacterias than community-acquired pneumonia? 

*Antibiotic choice as keeping in mind the local resistance pattern and doxycycline was initiated once scrub typhus was confirmed *

6. Why introduction of nebulized bronchodilators and inhaled corticosteroids treatment? There are not recommended of ARDS, may it an interesting treatment in case of *O. tsutsugamushi *pulmonary infection? 
*Nebulised bronchodilators and ICS were used as patient had occasional wheezing, not a known case of OAD. *
7. Low molecular weight heparin therapy seems to have been introduced according to the existence of a pro-inflammatory status (Elevated levels of C-reactive protein and D-dimer). With a NIV support responsible of a bed rest in a pubescent patient, is it not the standard care? Or is it a specific treatment in case of severe *O. tsutsugamushi* infection? 
*This was used as standard care and not specific to scrub typhus*
8. The conclusion could reinforce that Interstitial pneumonia images should make perform an ELISA test to search scrub typhus and introduce appropriate antibiotic treatment early in case of documentation and in such cases the treatment of *O. tsutsugamushi *is based on doxycycline or chloramphenicol, which are not the standard treatment in case of pneumonia. 

*Yes I agree, with you a detailed fever profile should be done for patients presenting with interstial pneumonias.*
